# Knowledge and Awareness among Parents and General Dental Practitioners regarding Rehabilitation with Full Coverage Restoration in Children: A Multi-centric Trial

**DOI:** 10.5005/jp-journals-10005-1359

**Published:** 2016-06-15

**Authors:** Aman Moda, Gyanendra Saroj, Swati Sharma, Basant Gupta

**Affiliations:** 1 Professor, Department of Pedodontics and Preventive Dentistry, Goenka Research Institute of Dental Science, Gandhinagar, Gujarat India; 2 Associate Professor, Department of Pedodontics and Preventive Dentistry, Maulana Azad Institute of Dental Science, Delhi, India; 3 Reader, Department of Pedodontics and Preventive Dentistry, Budha Institute of Dental Sciences and Hospital, Patna, Bihar, India; 4 Reader, Department of Prosthodontics, Jaipur Dental College, Jaipur Rajasthan, India

**Keywords:** Full coverage restorations, General dental practitioners, Knowledge, Parents.

## Abstract

**Aims:** The aim of this study was to evaluate the knowledge and awareness among parents and general dental practitioners regarding rehabilitation with full coverage restoration in children following pulp therapy.

**Materials and methods:** A multiple choice questionnaire was given to 1,000 parents and 400 general practitioners in this multicentric trial. The questionnaire assessed their beliefs, knowledge regarding care of primary teeth, assessment of treating children, and knowledge regarding importance of primary teeth. All the questionnaires were then compiled and statistically analyzed using Statistical Package for Social Sciences (SPSS) software.

**Results and discussion:** 53% parents did not know the importance of primary teeth and 73% parents also thought that no treatment is possible for pulpally involved primary teeth. 20% parents believed that root canal treatment can be possible for children and only 10% knew about full coverage restorations. 40% of the general dentists felt that the best treatment in the case of primary necrotic teeth is extraction and only 13% knew about stainless steel crowns. 62% of general dental practitioners pointed out patients’ noninterest in providing crowns whereas 68% parents reported non-information by dentists.

**Conclusion:** Both parents and general dental practitioners have incomplete and inadequate knowledge regarding full coverage restorations, and we need to improve the knowledge and dental awareness of the parents and the general dental practitioners.

**How to cite this article:** Moda A, Saroj G, Sharma S, Gupta B. Knowledge and Awareness among Parents and General Dental Practitioners regarding Rehabilitation with Full Coverage Restoration in Children: A Multi-centric Trial. Int J Clin Pediatr Dent 2016;9(2):177-180.

## INTRODUCTION

The level of oral health awareness and strategies for primary tooth care in developing nations is much lower as compared with Western nations. The care of primary teeth, especially their rehabilitation, is still an unexplored enigma in these countries. Since parents are the primary caregivers who are responsible for the well-being of their children’s teeth and also promoting oral health in them, an evaluation of their knowledge becomes essential.^1^ As general practitioners form a major component of dental practitioners in the urban and rural population, their knowledge and awareness regarding rehabilitation of primary teeth is a major unexplored area.

The endodontic treatment of primary teeth does provide symptom relief and masticatory rehabilitation to the patient, but just completion of endodontic procedure does not guarantee long-term success. It has been proved in many studies that teeth that have not been restored coronally following endodontic treatment have six times more chances of failure.^2^

The concept of full coverage restoration following an endodontic procedure in the case of permanent teeth is well established and well promoted by dental practitioners. However, the essence and necessity of full coverage restorations in post-endodontic primary teeth is still lagging. The aim of this study was to evaluate the knowledge and awareness among parents and general dental practitioners regarding rehabilitation with full coverage restoration in children.

## MATERIALS AND METHODS

This study was carried out on a multi-city level so as to gather a combined opinion of various cultural variations across northern India. A multiple choice questionnaire comprising 8 questions was handed out to 1,000 parents (250 in each center) and 400 general practitioners (100 in each center) in English as well as Hindi. One of the principal investigators was always present during the filling of the form so as to answer any queries of the respondents. The questionnaire pertaining to parental knowledge assessed their beliefs and knowledge regarding care of primary teeth, effect of caries, and attitudes. The form given to the general dental practitioner pertained more to his knowledge and assessment of treating children and his knowledge regarding importance of primary teeth. All the questionnaires were then compiled and statistically analyzed using Statistical Package for Social Sciences (SPSS) software.

## RESULTS

The results have been summarized in the forms below.

*Form I:* Parental knowledge and awareness regarding rehabilitation with full coverage restoration

**Table:** Importance of primary teeth

*Answer*		*Responses*		*Percentage*	
Primary teeth are important				47	
Primary teeth not important				50	
Do not know				03	

**Table:** Can pulpally involved primary teeth be treated?

*Answer*		*Responses*		*Percentage*	
Yes				20	
No				73	
Do not know				07	

**Table:** When do you visit your dentist?

*Answer*		*Responses*		*Percentage*	
Every 6 months				05	
During onset of pain symptoms				80	
When see decayed teeth				15	

**Table:** Choice of dentist

*Answer*		*Responses*		*Percentage*	
Family dentist				40	
General practitioner near home				40	
Specialist				20	

**Table:** Can root canal treatment be done for primary teeth?

*Answer*		*Responses*		*Percentage*	
Yes, can be done				20	
No, cannot be done				50	
Extraction is only treatment				30	

**Table:** Can crowns/full coverage restorations be given for primary teeth?

*Answer*		*Responses*		*Percentage*	
Yes, can be done				10	
No, cannot be done				60	
Can be done for adults only				30	

**Table:** Is it important to use crowns after pulpal treatment in primary teeth?

*Answer*		*Responses*		*Percentage*	
Yes				02	
No				78	
Do not know				20	

**Table:** Reason for non-usage of crown in primary teeth

*Answer*		*Responses*		*Percentage*	
Not informed by dentist				68	
Costly				15	
Not required as milk teeth will shed				17	

*Form II:* Knowledge and awareness regarding rehabilitation with full coverage restoration in general dental practitioners

**Table:** Total experience in dental practice

*Answer*		*Responses*		*Percentage*	
1-5 years				32	
5-10 years				40	
More than 10 years				28	

**Table:** Average no. of pediatric dental patients treated daily

*Answer*		*Responses*		*Percentage*	
1-5 daily				16	
1-5 in a week				79	
1-5 in a month				05	

**Table:** Pediatric dental patients referred

*Answer*		*Responses*		*Percentage*	
To family dentists				14	
To pediatric dentists				10	
Not referred				76	

**Table:** What is the best treatment for pulpally involved primary teeth?

*Answer*		*Responses*		*Percentage*	
Extraction				40	
RCT				29	
No treatment is required as they are milk teeth				31	

**Table:** Is it important to use crowns after pulpal treatment in primary teeth?

*Answer*		*Responses*		*Percentage*	
Yes				33	
Not required				25	
Crowns only for permanent teeth				42	

**Table:** What is the best type of full coverage restoration in primary teeth?

*Answer*		*Responses*		*Percentage*	
Stainless steel crowns				13	
Lab fabricated				77	
Do not know				10	

**Table:** Are aesthetic crowns for primary teeth available?

*Answer*		*Responses*		*Percentage*	
Yes				11	
No				50	
Do not know				39	

**Table:** Reason for non-usage of crowns in primary teeth

*Answer*		*Responses*		*Percentage*	
Patient not interested				62	
Cost factor				18	
Do not have knowledge				20	

## DISCUSSION

The main objective of this study was to evaluate the knowledge and awareness of the parents and general dental practitioners. The quality of oral health care in children is directly proportional to the knowledge of their parents.^3^ Hence, it is important to understand the parental perception about the importance of primary teeth.^1,4^ Sarnat et al^5^ reported that the more positive the mother’s attitude towards dental health the better is the child’s oral hygiene. In the present study, 53% parents did not know the importance of primary teeth and 73% parents also thought that no treatment is possible for pulpally involved primary teeth. This is in conjunction with the studies of Nagaveni et al^1^ and Lahti et al,^6^ which also observed similar findings. 80% parents visited the dentist on the patients’ complaints of pain and associated symptoms and 40% preferred their family doctor or general practitioner as compared with a specialist.^3,4^ Another interesting parameter of this study was that only 20% parents believed that root canal treatment could be possible for children. When enquired about crowns and their usage, only 10% knew about full coverage restorations and even lesser parents 2% knew about the effect of full coverage restoration on endodontically treated primary teeth.^3^ The most plausible reason provided for this was non-information by the dentist followed by the cost factor. A similar study by Nagasiri et al reported the patient’s knowledge of about 20% in permanent teeth.^7^

The reason for inclusion of general dental practitioners in this study was to evaluate their awareness regarding full coverage restorations in post-endodontically treated primary teeth as they were the first ones to encounter the patients. Their answers were assessed according to experience, number of pediatric patients seen, and the knowledge. 40% of the general dentists felt that the best treatment in case of primary necrotic teeth is extraction. Hussain et al^8^ also evaluated the same in their study. Only 33% of the general dental practitioners realized the importance of full coverage restoration after primary pulp therapy out of which only 13% knew about stainless steel crowns. Most of the practitioners who answered these were of relatively younger generation. The main reason for this could be inclusion of such advancements in the recent undergraduate curriculum. McKnight-Hanes et al^9^ compared the treatment recommendations for the primary teeth and concluded that more general dental practitioners recommended restorations whereas pediatric dentists recommended use of stainless steel crowns followed by pulp therapy. 62% of general dental practitioners pointed out patient’s noninterest in providing crowns whereas cost and lack of knowledge were the secondary reasons.

The goal of endodontic therapy, especially in primary teeth, is to maintain the stability of teeth in the dental arch and improve aesthetics and functions. According to Morgano et al,^10^ the strength of endodontically treated teeth is directly proportional to the remaining Dentin. As the dentinal strength is greatly reduced in endodontically treated primary teeth, it is mandatory for us to use full coverage restorations to have long-term prognosis. Most of the studies reveal that root-filled teeth should always be restored properly as their clinical success depends more on the final restorations rather than the endodontic treatment.^11^

## CONCLUSION

The present study was initiated keeping in mind the value of full coverage restorations in endodontically treated primary teeth, and the reason for including the parents and the general dental practitioners was that they are service providers and the first available dentists to be shown the case. The findings of this study reveal that both the parents and the general dental practitioners have incomplete and inadequate knowledge regarding full coverage restorations. The most important reason given by the parents was non-information by the dentists and the most sought after reason of the general dental practitioners was noninterest of the parents. These two findings are highly co-related with each other, thus creating a circle of defiance regarding dental awareness. The conclusion drawn from this study is that we need to improve the dental awareness of the parents as well as update the knowledge of the general dental practitioners so that they can inculcate a positive dental attitude in the patients and parents.
